# The Use of Biologic Mesh for Repair of Perianal Fistulas

**DOI:** 10.7759/cureus.64675

**Published:** 2024-07-16

**Authors:** Nicholas Gemma, Kevin McMahon, McKenzie Clapp, Truong Ma, Erica Laipply

**Affiliations:** 1 General Surgery, Summa Health, Akron, USA; 2 Surgery, Summa Health, Akron, USA; 3 Colorectal Surgery, Summa Health, Akron, USA

**Keywords:** anorectal diseases, colorectal surgeon, biologic mesh, anorectal fistula, perianal fistula

## Abstract

This is a case series of three patients who presented with complex anorectal fistulas. Each patient underwent repair of complex anorectal fistulas with biologic mesh. We will discuss each case and our institution’s experience with this relatively new technique.

This case series demonstrates the use of biologic mesh for the repair of complex anorectal fistulas. Three patients are discussed who underwent repair of perianal fistulas using ACell mesh by two separate surgeons. We will discuss the rationale for offering this treatment, as well as the advantages and disadvantages. The use of biologic mesh in perianal fistulas is a relatively new topic that needs further investigation.

Perianal fistulas can be difficult to manage for both patients and surgeons. There are many options for repair, ranging from simple to complex. Biologic mesh for complex fistulas may be a useful option to avoid the morbidity of more complex repairs, such as flaps.

## Introduction

Perianal fistulas or anorectal fistulas are defined as epithelialized tracks involving the anus or rectum. When an abscess forms in the anorectal region, it either ruptures or is drained. Approximately one-third of patients with anorectal abscesses will develop fistulas [1]. The true prevalence of anorectal fistulas is unknown but it is a relatively common issue in the world of colorectal surgery and can result in significant morbidity for the patient. There are many causes of anorectal fistulas, including obstetric injury, Crohn’s disease, radiation, infection, or cryptogenic abscesses [2]. Anorectal fistulas are classified both by the relationship to anal sphincters and degree of complexity. Fistulas can be superficial, intersphincteric, trans-sphincteric, extrasphincteric, or suprasphincteric. Complex fistulas are defined as those that involve greater than 30% of the external anal sphincter, suprasphincteric, or those relating to Crohn’s or malignancy [2]. Diagnosis is usually made by physical examination or examination under anesthesia with hydrogen peroxide. However, imaging such as MRI may occasionally be necessary for more complex fistulas. Management of anorectal fistulas is dependent on multiple factors, including etiology and classification [1]. The basic principles of management involve eliminating the fistula while maintaining the function of the sphincter. Simple fistulas may only require sphincterotomy. More complex fistulas, such as those involving more than one-third of the sphincter complex, may involve multiple interventions and complex surgical procedures with high morbidity [2]. The goal in management should be a step-wise approach to eradicate the fistula while limiting morbidity and preserving sphincter function [1]. This case series will discuss using a biologic mesh in the treatment of complex fistulas as an option to avoid more morbid surgeries. The use of biologic mesh is a relatively new topic in the world of colorectal surgery [3]. Thus far, results have been promising but more investigation is needed.

## Case presentation

Three cases will be presented describing our experience with this technique. Consent was not needed to be obtained from the patients because this is a limited case series without patient identifiers.

**Figure 1:**
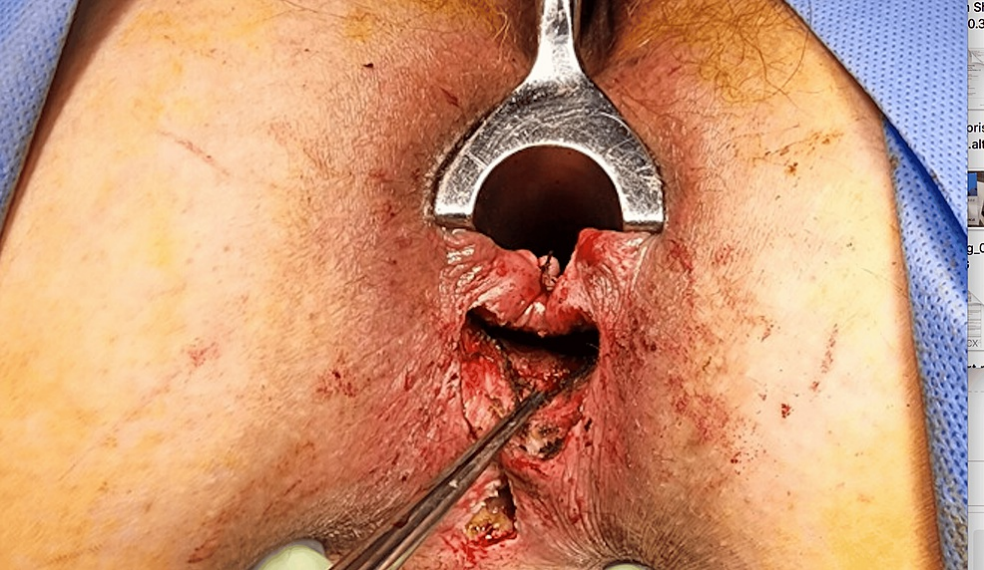
Intraoperative photo 1, the identification of the fistulous track. Patient in supine lithotomy. The bottom of the image is posterior and the top of the image is anterior. The figure shows a curvilinear incision made for the identification of the fistulous track in Patient A.

**Figure 2:**
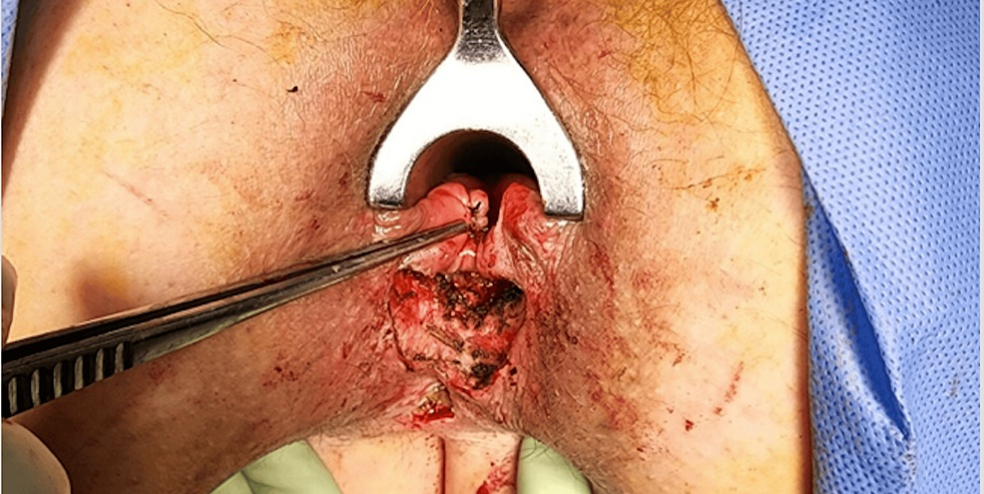
Intraoperative photo 2, debridement of the fistulous track for better identification. The figure shows the debridement of the fistulous track in Patient A for better visualization.

**Figure 3:**
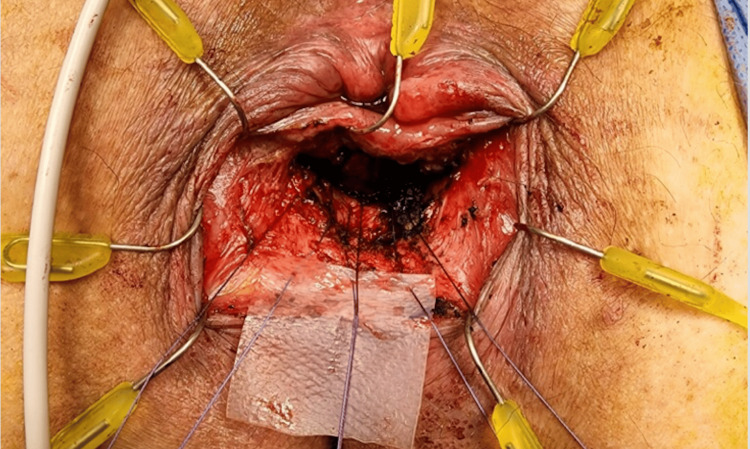
Intraoperative photo 3, mesh placement. The figure shows the fistulous track closed with Vicryl sutures. A piece of biologic ACell mesh is being placed over the fistula and parachuted in with Vicryl suture.

**Figure 4:**
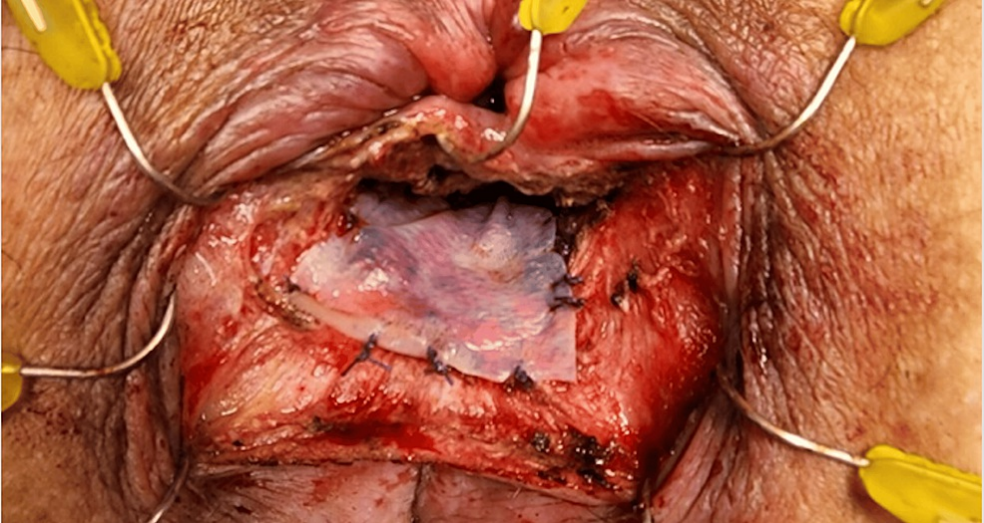
Intraoperative photo 4, mesh placement. The figure shows the biologic mesh sutured into place over the fistula. The curvilinear incision will then be closed so the mesh is not exposed.

Patient A is a 47-year-old female with no significant comorbidities who presented to the colorectal clinic due to concern for perianal fistula after colonoscopy by a referring provider. She was taken for a rectal examination under anesthesia for further evaluation and was found to have a trans-sphincteric fistula in the posterior midline involving greater than one-third of the sphincter complex. A seton was placed. Months later, she was brought for a rectal examination under anesthesia for further evaluation and definitive repair. The internal opening of the fistula was approximately 5 mm. A curvilinear incision was made in the posterior anal canal at the level of the intersphincteric groove and a dissection was made into the intersphincteric space, as shown in Figure 1. The fistula was identified in the intersphincteric space and dissected out, as shown in Figure 2. The fistula was then ligated. The defect was closed on both sides using 3-0 running and interrupted Vicryl sutures. Hydrogen peroxide was used and the repair was found to be intact. We then decided to use a 3 × 3 cm piece of ACell mesh in the intersphincteric space to provide coverage over the repair. The mesh was secured with 3-0 Vicryl sutures, as shown in Figure 3 and Figure 4. The incision was closed and the procedure was complete.

Patient B is a female in her early 20s with Crohn’s disease. She had been taken for a prior rectal examination under anesthesia for anal fissures and fistulas. She underwent fissurectomy and sphincterotomy for anterior and posterior anal fissures. She had also undergone incision and drainage of perianal abscess and placement of seton for anterior anal fistula by another provider. She was referred to the colorectal clinic for evaluation of a complex fistula. She was taken to the operating room for a rectal examination under anesthesia. She was found to have an anterior fistula, which was probed and determined to be transsphincteric. A curvilinear incision was made in the intersphincteric groove and a plane was dissected into the intersphincteric space. The fistulous tract was identified and dissected. The fistula was then ligated. The defects were closed using 3-0 Vicryl until no leakage of hydrogen peroxide was detected. A 3 × 4 cm piece of ACell mesh was used over the defect and secured using 3-0 Vicryl stitches. The incision was closed and the procedure was complete.

Patient C is a healthy 45-year-old female who initially presented for the incision and drainage of a large perineal abscess by gynecology. This abscess extended from the vagina into the rectum. Colorectal surgery was consulted postoperatively and the patient did require a diverting loop ileostomy for fecal diversion considering the size of the rectovaginal fistula. The patient was allowed time to heal while diverted. However, the fistula would not heal so operative repair was indicated. Multiple options were offered to the patient, including allowing more time for healing, primary repair with biologic mesh, endorectal advancement flap (which was considered not feasible due to the location of the fistula), and referral for more complex flap reconstruction. The patient agreed to primary repair with biologic mesh. The procedure was done in conjunction with gynecology. The rectovaginal fistula was identified intraoperatively with a probe. A small curvilinear incision was made in the intersphincteric groove. A small space around the fistula was dissected free. The fistula tract was ligated and closed on both sides using multiple 3-0 Vicryl interrupted sutures. A 2 × 2 cm piece of ACell mesh was then fixed in position over the repair and the incision was closed.

All three patients followed up in the office one month postoperatively. History and physical examinations were performed including anoscopy. Patients A and B are doing well with no signs of complication. Patients A and B both experienced postoperative pain but no incontinence or leakage. The mesh is not visible on examination and incorporates with the tissue. Patient C was taken back to the operating for a rectal examination under anesthesia for evaluation of another fistula. There was a small dehiscence to the prior repair. She will continue to follow up in the office every three to six months for an anorectal examination and consideration of ileostomy reversal. All three cases have been performed in the past two years so further follow-up is needed.

## Discussion

In the world of colorectal surgery, perianal fistulas are a common problem but can be difficult to manage. The goal of repair is to eliminate the fistula while preserving the sphincter [2]. In some cases, this can be challenging and requires complex repair. Treatment options range from simple repairs such as fistulotomies to complex, morbid operations such as myocutaneous flaps [1]. The goal should be to start with the simplest, least morbid option and work toward more complex repairs if needed. A repair using biologic mesh seems to be a reasonable option for complex fistulas [3]. This is a relatively new treatment modality both in the world of surgery and in our institution. Results have been promising thus far, but more cases and experience are needed for further analysis [3].

There has been literature published in the past decade regarding the use of biologic mesh for the treatment of perianal fistulas. However, the data is not sufficient and the results have varied widely. Some reports refer to this technique as the BioLIFT because it incorporates biologic mesh into the ligation of intersphincteric tract (LIFT) technique. A comparison of different management options by [1] Zahra et al. revealed that some studies report success rates of up to 95% with the use of biologic mesh in conjunction with the LIFT procedure. Other studies have reported success rates of around 50%. A retrospective cohort study [3] by Zwiep et al. showed that the BioLIFT technique had significantly higher success rates in eradicating complex fistulas compared to the standard LIFT procedure. There is also variation in technique regarding the use of mesh. Some have used the mesh as a plug while others as an overlay. Our experience has only involved overlay. The use of mesh in recurrent fistulas has been less successful according to current data [3]. The fact is that the use of biologic mesh for the repair of fistulas is a newer treatment option that requires more investigation. A 2020 retrospective analysis by Tsang et al. used the BioLIFT technique in patients who had a prior failed LIFT procedure and achieved healing rates of 80% without associated complications [4]. A 2013 study by Chew et al. retrospectively reviewed predominantly female patients (67%) with trans-sphincteric fistulas who underwent LIFT and BioLIFT and achieved a primary healing rate of 63%. A predictor of failure to heal included anterior fistulas. Multiple patients who failed LIFT subsequently underwent BioLIFT successfully [5]. This strengthens the argument for the use of biologic mesh before proceeding to more morbid operations. 

There are a few potential disadvantages to using biologic mesh for the treatment of fistulas. The first is cost. Cost varies depending on the product but it is definitely a consideration, given other techniques can be done without the added cost of mesh. The placement of the mesh also adds operative time. Additionally, this technique involves the placement of a foreign object into the patient. There are potential complications of leaving a foreign body in a patient including persistent fistula. More data is needed to determine if the benefits outweigh the risks.

## Conclusions

The goal of this case series is to further highlight the use of biologic mesh in the repair of perianal fistulas and promote further investigation. Perianal fistulas can be challenging for a surgeon to manage and they can significantly affect a patient’s quality of life. The role of biologic mesh may be to provide a repair for complex fistulas with less morbidity than more complex procedures such as flap. The use of biologic mesh in fistula repairs is relatively new to both our institution and the surgical community. Our experience and the current data are promising but there needs to be further investigation into the efficacy of biologic mesh use.
